# Biomaterials-based formulations and surfaces to combat viral infectious diseases

**DOI:** 10.1063/5.0029486

**Published:** 2021-02-09

**Authors:** Sushma Kumari, Kaushik Chatterjee

**Affiliations:** Department of Materials Engineering, Indian Institute of Science, Bangalore 560012, India

## Abstract

Rapidly growing viral infections are potent risks to public health worldwide. Accessible virus-specific antiviral vaccines and drugs are therapeutically inert to emerging viruses, such as Zika, Ebola, and severe acute respiratory syndrome coronavirus 2 (SARS-CoV-2). Therefore, discovering ways to prevent and control viral infections is among the foremost medical challenge of our time. Recently, innovative technologies are emerging that involve the development of new biomaterial-based formulations and surfaces endowed with broad-spectrum antiviral properties. Here, we review emerging biomaterials technologies for controlling viral infections. Relevant advances in biomaterials employed with nanotechnology to inactivate viruses or to inhibit virus replication and further their translation in safe and effective antiviral formulations in clinical trials are discussed. We have included antiviral approaches based on both organic and inorganic nanoparticles (NPs), which offer many advantages over molecular medicine. An insight into the development of immunomodulatory scaffolds in designing new platforms for personalized vaccines is also considered. Substantial research on natural products and herbal medicines and their potential in novel antiviral drugs are discussed. Furthermore, to control contagious viral infections, i.e., to reduce the viral load on surfaces, current strategies focusing on biomimetic anti-adhesive surfaces through nanostructured topography and hydrophobic surface modification techniques are introduced. Biomaterial surfaces functionalized with antimicrobial polymers and nanoparticles against viral infections are also discussed. We recognize the importance of research on antiviral biomaterials and present potential strategies for future directions in applying these biomaterial-based approaches to control viral infections and SARS-CoV-2.

## I. INTRODUCITON

In the current world, viral infections pose a formidable challenge to human life. Virus-triggered lower respiratory infections, for example, influenza, pneumonia, and bronchitis, are the leading cause of millions of deaths worldwide and a rise in costs of healthcare.^1^ New infectious pathogens, such as the Zika virus, Ebola virus, pandemic influenza, West Nile virus, severe acute respiratory syndrome (SARS), and SARS-CoV-2, have emerged in recent years. The lack of immediate effective prevention or treatment has caused some of the most dramatic and deadly disease pandemics in recent human history, with a severe impact on public healthcare systems and a downturn in socio-economic growth.^2,3^ The emerging threat of uncontrolled viral diseases has set off an international drive to develop innovative platforms with a very wide variety of concepts and approaches for the management and treatment of infectious diseases.

To date, vaccination remains very effective in stimulating protective immune responses against infections. Successful vaccination against common diseases, such as diphtheria, measles, mumps, pertussis, polio, tetanus, rubella, hepatitis B, meningitis, and smallpox, has contributed greatly in reducing the morbidity and mortality of humans by 97%–99%.^4^ Typically, conventional vaccines derived from live attenuated pathogens and inactivated viruses, recombinant proteins, and synthetic peptides are being used to elicit protective immune responses against pathogens. Despite several merits, these vaccines also suffer from their own drawbacks for adverse reactions, low stability in the bloodstream, and poor effectiveness against infectious diseases. To overcome these limitations, antigenic subunits, natural or recombinant proteins, are formulated with immunologic adjuvants to stimulate and enhance the immunogenicity of antigens. Inorganic salt-based aluminum hydroxide (alum) is a widely used adjuvant in the market, but the application is limited by allergic reactions at the injection site with the formation of subcutaneous nodules and mild toxicity in large doses.

Recent advances in biotechnology and nanotechnology have encouraged the engineering of biomaterial-based nanocarriers for next-generation vaccines and adjuvant formulations.^5,6^ Synthetic and natural polymeric particles, lipids, self-assembled proteins, virus-like-particles, and inorganic particles are all promising nanocarriers and have shown great potential in the delivery of therapeutic agents to induce appropriate immune responses against targeted pathogens. Potential advantages of nanocarrier-based delivery systems include (1) smaller particle size (facilitates drug delivery into anatomically privileged sites), (2) high surface area to volume ratios (affords formulations with high drug payload), (3) surface functionalization (provides stable structures for drug encapsulation), (4) tunable surface charge (promotes efficient cellular uptake), and (5) biomimetic characteristics (mimics pathogen features).^7^ Here, biomaterials technology provides a platform for co-encapsulation of antigens and immunostimulants and cargo protection against enzymatic degradation and allows targeting of antigen-presenting cells (APCs) and controlled vaccine release kinetics.^5^ Altogether, these properties enable additional control over programming protective immune responses with adjuvants inducing humoral and cellular immunity without adverse reactions to vaccines.

Developing self-disinfecting surfaces (inactivation of microbes on contact) is a step forward to control outbreaks and transmission of enteric and respiratory viruses.^8^ Contaminated surfaces or fomites are an important source of transmission of viral infections since a variety of pathogens are deposited on these surfaces and can be easily transmitted to whoever else contacts the surface. For example, in the event of new epidemics or pandemics, contaminated surfaces have played a significant role in the rapid spread of viral infection, specifically in crowded places, public transport systems, indoor establishments, business offices, and healthcare facilities, and have shown a direct and dramatic effect on morbidity and mortality around the world.^9,10^ Notably, interruption of indirect transmission of pathogens can be accomplished with self-disinfecting surfaces by the application of antimicrobial coatings that will also bring down the labor and time spent in decontaminating the surfaces. In this quest, both conventional methods and advanced surface modification techniques have been used to kill or efficiently reduce the attachment of pathogenic microbes on different material surfaces. Nowadays, intensive research has been focused on developing an effective antimicrobial surface using both physical approaches (such as surface topography and surface treatment) and chemical approaches (such as surface functionalization, polymerization, and derivatization).^11–13^

**FIG. 1.**
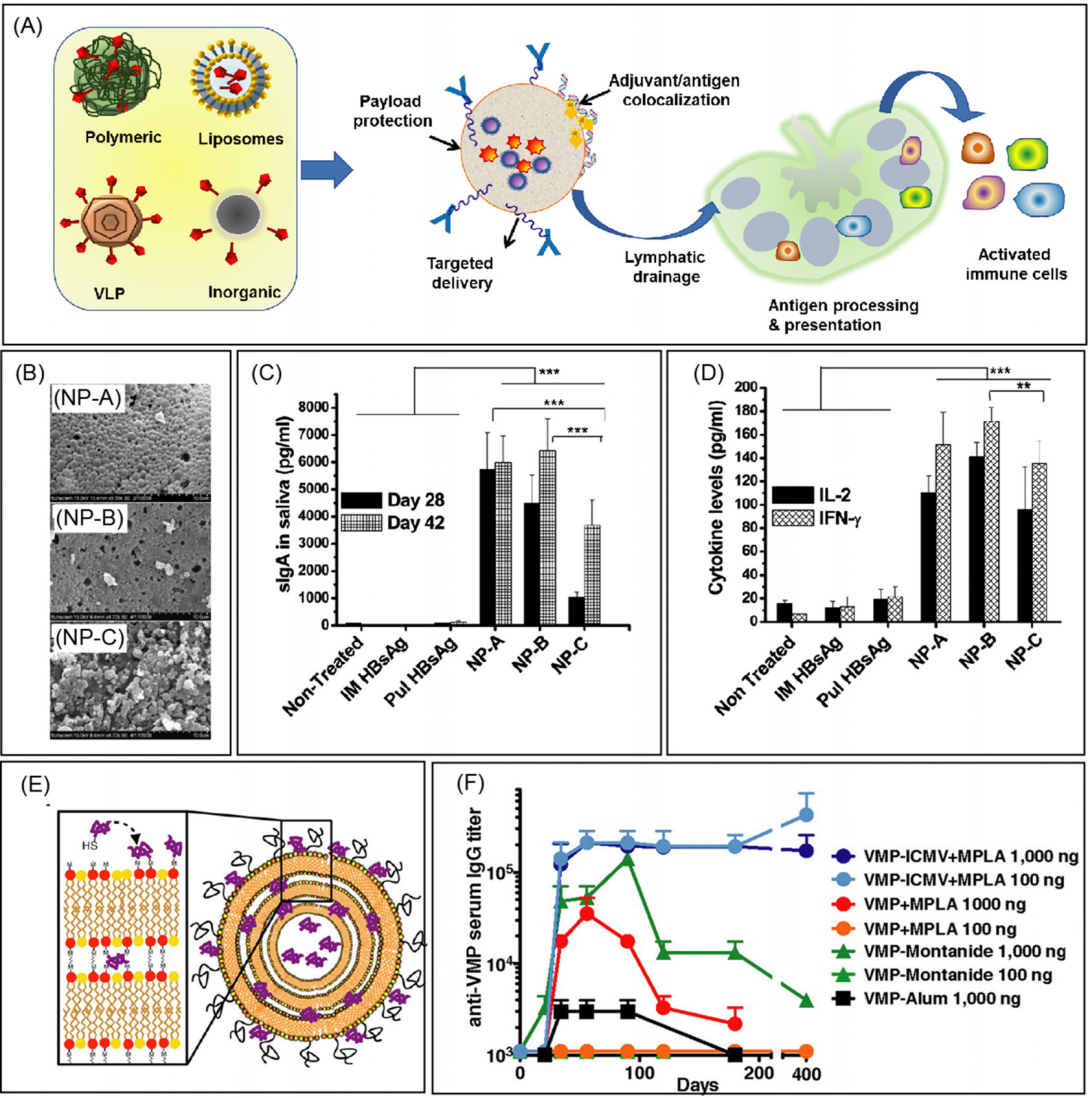
Vaccine nanotechnology. (a) Schematic illustration of the nanoparticle-based vaccine formulations. Nanoparticles present many advantages, such as protection of encapsulated payloads and bioactivity, codelivery of the antigen and adjuvant to immune cells, and surface functionalization with ligands for cell-targeted delivery. The small size of nanoparticles enables efficient lymphatic transport and facilitates antigen presentation for the stimulation of the immune system to induce both humoral and cellular immunity. (b) SEM images of three formulations of poly(l-lactic acid) (PLA) and poly(lactic-co-glycolic acid) (PLGA) nanoparticles containing a fixed amount of hepatitis B surface antigen (HBsAg), where PLA, PLGA 85/15, and PLGA 50/50 were designated as NP-A, NP-B, and NP-C. Nanoparticulate formulations were prepared using PLA or PLGA by the w/o/w double emulsion solvent evaporation method. (c) Mucosal immune response profile showing statistically significantly increased sIgA levels in the bronchoalveolar lavage (BAL) fluid of rats immunized with three formulations on days 28 and 42. (d) Nanoparticulate formulations show a significant increase in both Interferon-γ (IFN-γ) and interleukin-2 (IL-2) levels in spleen homogenates of rats immunized with three formulations at the end of 6 weeks. (b)–(d) Reproduced with permission from Thomas *et al.*, Mol. Pharm. **8**, 405 (2011) Copyright 2011 American Chemical Society. (e) Schematic illustration of Interbilayer-crosslinked multilamellar vesicles (ICMVs) with surface-conjugated VMP001 via coupling of cysteine residues with maleimide MAL-functionalized lipids. (f) VMP001-ICMV vaccines elicit robust, durable antibody titers with significantly reduced antigen/adjuvant doses in mice. High titers of serum anti-VMP001 IgG sustained for more than 1 y following a prime and boost with as little as 100 ng of the malaria antigen. (e) and (f) Reproduced with permission from Moon *et al.*, Proc. Natl. Acad. Sci. U. S. A. **109**, 1080 (2012). Copyright 2012 National Academy of Sciences.

**FIG. 2.**
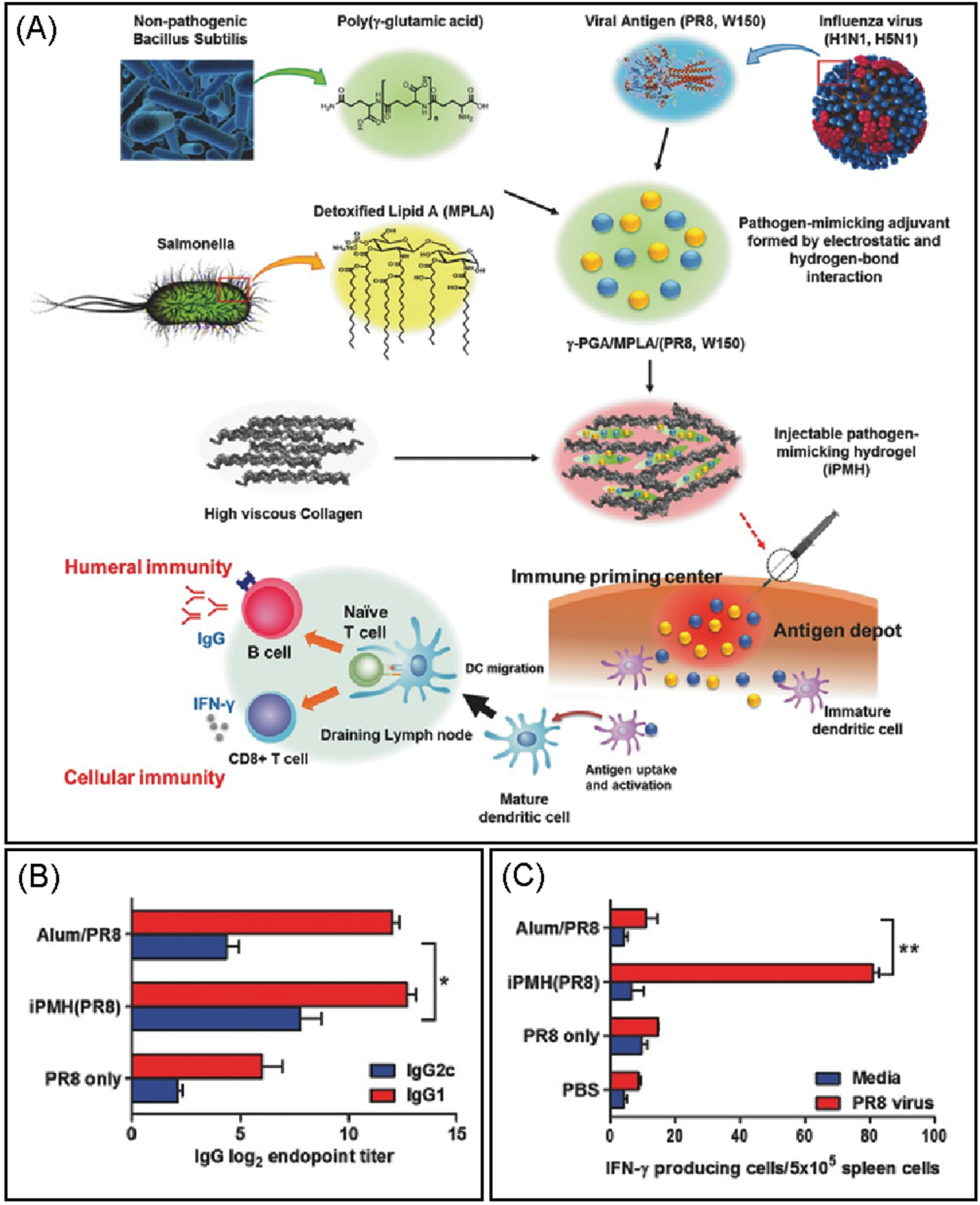
Schematic design of injectable pathogen-mimicking hydrogel (iPMH) and vaccination for antiviral immunity. (a) Pathogen-mimicking adjuvants were fabricated by the combination of hydrophobic immunostimulatory 3-O-desacyl-4′-monophosphoryl lipid A (MPLA) and viral antigens (H1N1, H5N1) with mixing poly(γ-glutamic acid) (γ-PGA). The γ-PGA/MPLA/antigen complex was transformed into an injectable hydrogel (iPMH) by the combination of collagens. At the injection site, the iPMH system functions as the pathogen-mimicking immune priming center and antigen depot that promotes the stimulation of the immune system to induce both humoral and cellular immunity. Influenza A/PuertoRico/8/34 (PR8, H1N1) virus-specific immune responses in vaccinated mice and their survival rates after a lethal challenge. (b) PR8 virus-specific IgG, IgG1, and IgG2c antibody titers, observed in C57BL/6 mice (*n* = 5), immunized intramuscularly with PR8 (0.5 *μ*g) only, iPMH(PR8), and Alum/PR8. (c) Cellular immune responses analyzed in the splenocytes for IFN-γ production by re-stimulation with the PR8 viral protein. Reproduced with permission from Noh *et al.*, Small **12**, 6279 (2016). Copyright 2016 Wiley‐VCH Verlag GmbH & Co. KGaA, Weinheim.

**FIG. 3.**
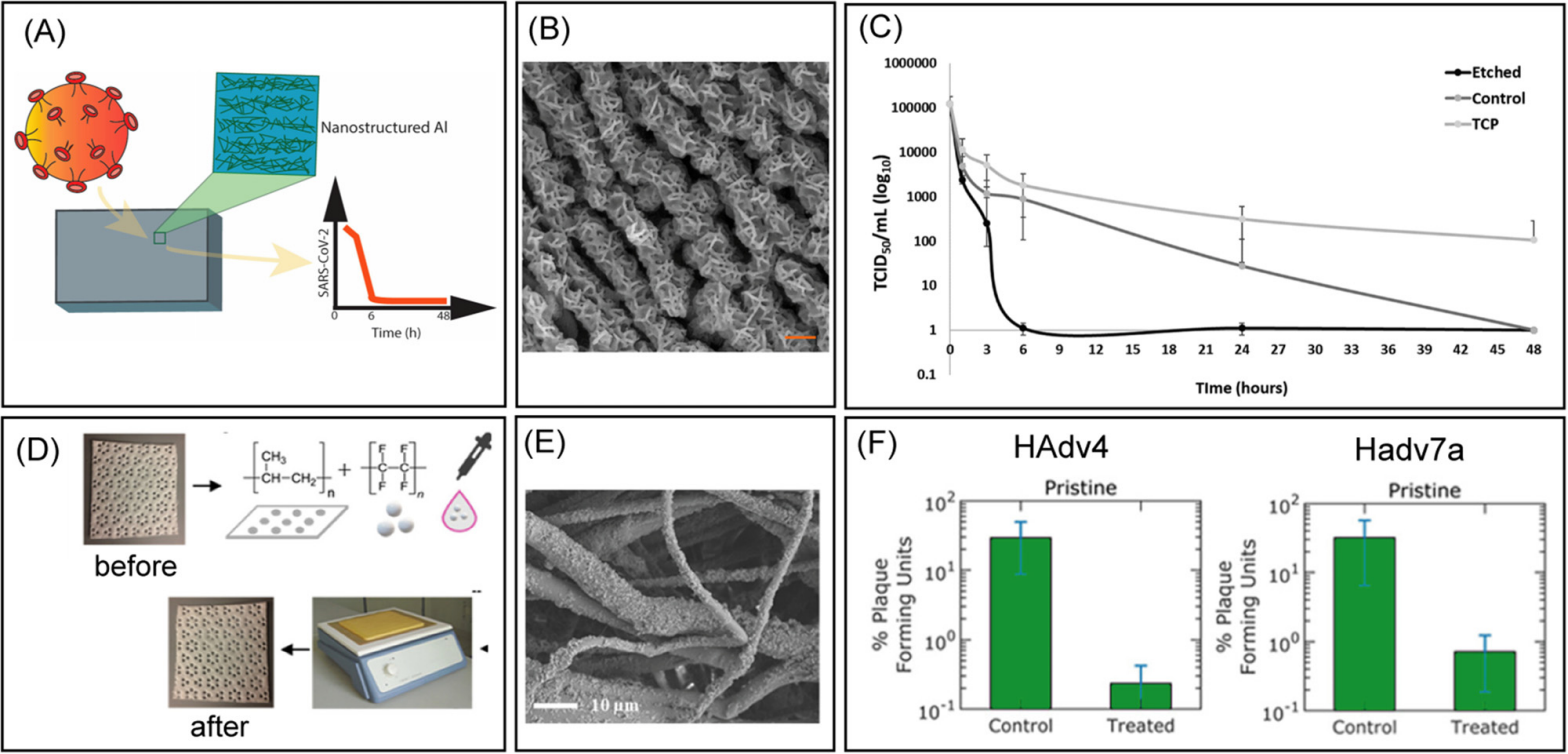
Anti-adhesive surfaces. (a) Schematic representations of the etched Al 6063 samples and interaction with SARS-Cov-2. (b) Scanning electron microscopy image (SEM) of the etched Al for nanostructured topography. Scale bar = 200 nm (c) Viability of SARS-CoV-2 on the surfaces of the etched (nanostructured) Al 6063 alloy, control Al 6063 alloy, and nonmetal surface tissue culture plates (TCPs) at different time intervals of 1, 3, 6, 24, and 48 h. The titers of viable viruses are expressed as TCID50/mL on a logarithmic scale. (a)–(c) Reproduced with permission from Hasan *et al.*, ACS Biomater. Sci. Eng. **6**, 4858 (2020). Copyright 2020 American Chemical Society. (d) A schematic illustrating the textile treatment method using drop casting and heat treatment processes. (e) SEM images showing the surface morphology of treated PP textiles. (f) Percent plaque forming units (PFU) of adenovirus HAdv4 and Hadv7a on control and treated samples after incubation. (d)–(f) Reproduced with permission from Galante *et al.*, ACS Appl. Mater. Interfaces **12**, 22120 (2020). Copyright 2020 American Chemical Society.

**FIG. 4.**
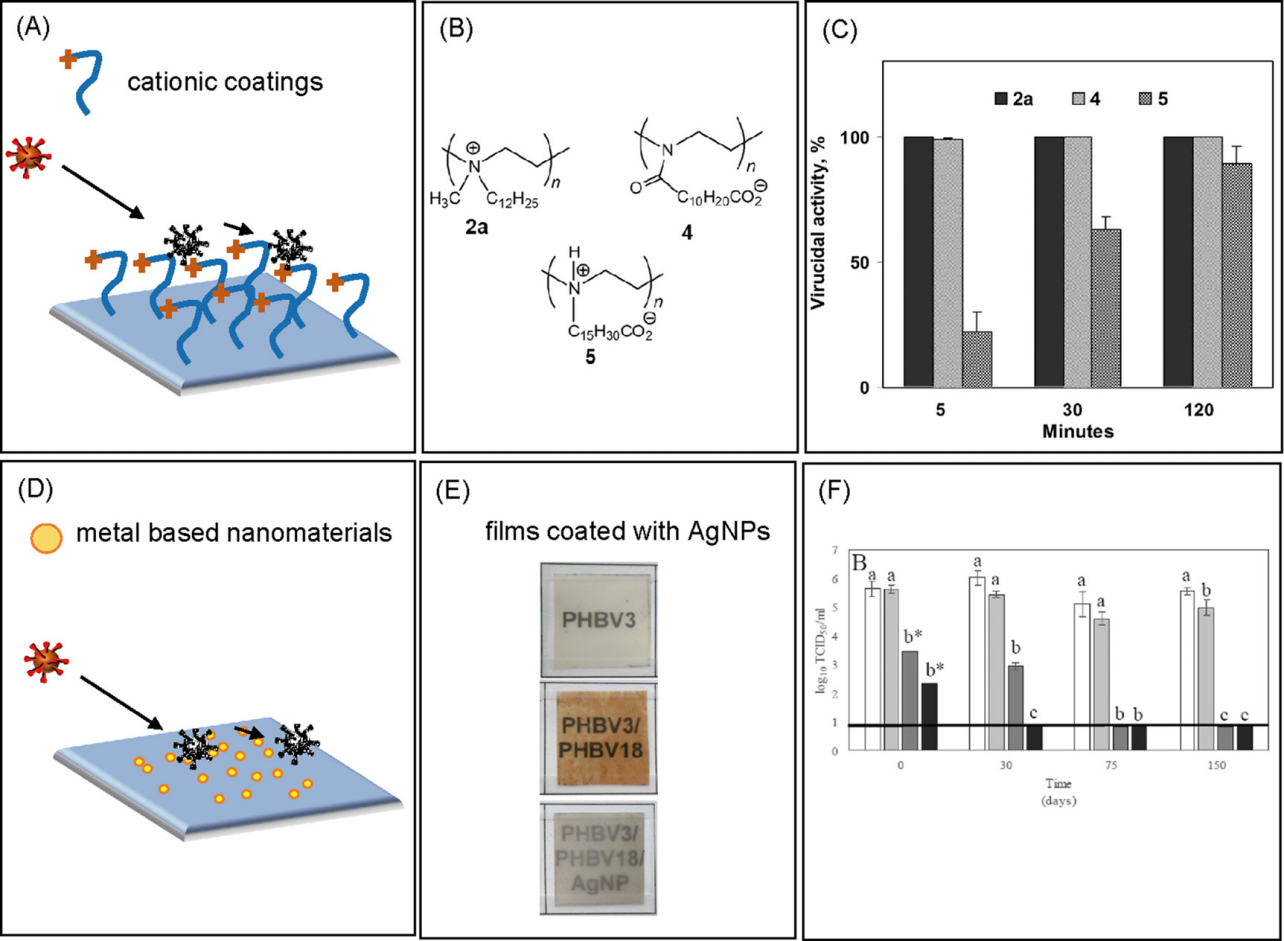
Antiviral surfaces. (a) Schematic representation of surface functionalization with cationic polymers. (b) Chemical structures of hydrophobic PEI derivatives. (c) The virucidal activity against influenza virus (WSN strain) of glass slides painted with **2a**, **4**, and **5** after different times of exposure at room temperature. (a)–(c) Reproduced with permission from Haldar *et al.*, Proc. Natl. Acad. Sci. **103**, 17667 (2006). Copyright (2006) National Academy of Sciences. (d) Schematic representation of surface functionalization with metal-based nanomaterials. (e) Contact transparency pictures of PHBV3, PHBV3/PHBV18, and PHBV3/PHBV18/AgNP. (f) Antiviral effect of silver nanoparticles at different concentrations (0, 2.1, 10.5 and 21 mg/L) on murine norovirus (MNV) over the storage time. (d)–(f) Reproduced with permission from Castro-Mayorga *et al.*, LWT-Food Sci. Technol. **79**, 503 (2017). Copyright (2017) Elsevier.

**FIG. 5.**
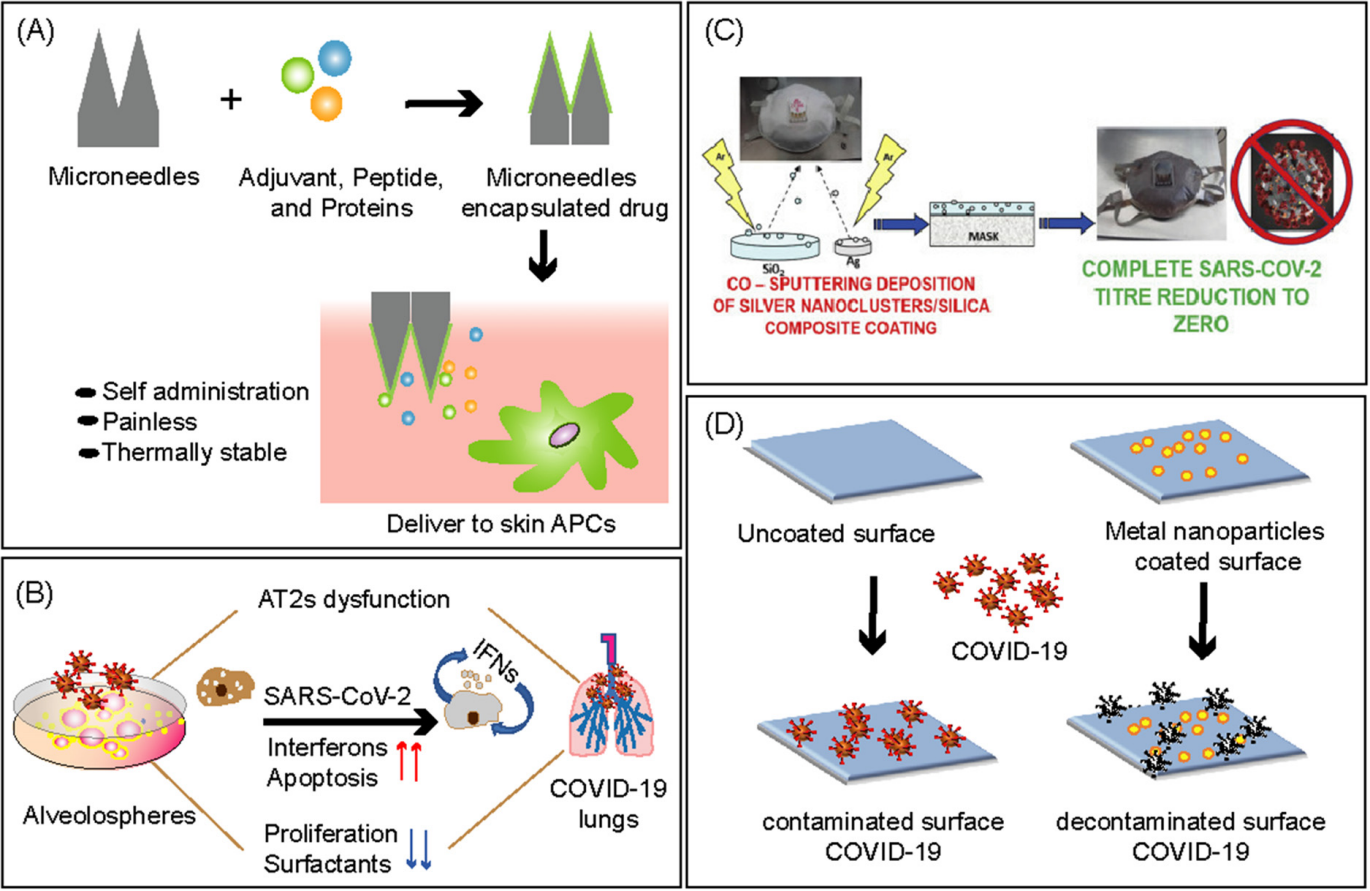
Biomaterial-based strategies against COVID-19 pandemic. (a) Schematic representation of the microneedle-based vaccine delivery system that increases the safety of patients by efficiently targeting skin-resident immune cells with an improved cellular immune response. (a) Reproduced with permission from Bookstaver *et al.*, Trends Immunol. **39**, 135 (2018). Copyright (2017) Elsevier Ltd. (b) Schematic representation of the human lung AT2 alveolosphere culture system. Cultured AT2s are conducive to SARS-CoV-2 infection and elicit the expression of transcriptome-wide changes that mirror COVID-19 histopathology, with upregulation of inflammatory responses, cell apoptosis, and downregulation of surfactant protein, leading to pneumocyte dysfunction. (b) Reproduced with permission from Katsura *et al.*, Cell Stem Cell **27**, 1 (2020). Copyright (2020) Elsevier Inc. (c) Disposable non-woven fabric-based facile FFP3 mask prepared by sputter coating with antimicrobial/virucidal silver nanocluster/silica composite solution. (c) Reproduced with permission from Balagna *et al.*, Open Ceram., **1**, 100006 (2020). Copyright (2020) WHO COVID. (d) Schematic representation to tackle COVID-19 through the application of biomaterial-based antimicrobial coatings using metal nanoparticles.

In the present review article, we have categorized and discussed how biomaterial-based technologies are currently being used for both minimizing the spread of the virus and curing viral infections and diseases. We will first review recent effort in biomaterial-based NPs to address the limitation of existing antiviral drugs and vaccines, which include particles made up of polymers, lipids, self-assembled proteins, and inorganic metal/metal oxides (Fig. 1). We have also reviewed scaffold-based strategies for fighting against infectious diseases (Fig. 2) and natural products with demonstrated antiviral properties against viral diseases. Next, we have discussed recent research effort on the next generation of antiviral surfaces designed through nanostructured surfaces, antiadhesive surfaces, and intrinsic antiviral materials (Figs. 3 and 4). Finally, we have discussed future prospects to further advance the biomaterial technologies against emerging infectious diseases and COVID-19 (Fig. 5).

## II. VACCINES AGAINST INFECTIOUS DISEASES

Vaccination is widely recognized as one of the most cost-effective means of controlling and combating infectious diseases. Vaccines have allowed the prevention and eradication of diseases such as smallpox, poliovirus, malaria, tetanus, and diphtheria.^14^ Typically, vaccines contain live-attenuated pathogens, killed pathogens, or recombinant proteins, which protect individuals by eliciting a specific immune response. However, live-attenuated vaccines suffer from safety concerns and have high potential to cause diseases in immunocompromised patients. On the other hand, virus-derived subunit vaccines and inactivated pathogen vaccines are poorly immunogenic and often require the use of immune-stimulating complexes as an adjuvant to boost vaccination.^15^ However, the application of several clinically tested adjuvants is limited by the early degradation of vaccine ingredients and the requirement of adequate doses to generate immunity.^16^

Currently available antiviral therapeutics can be categorized on the basis of their mechanisms of action such as reverse transcriptase inhibitors [a retrovirus, human immunodeficiency virus (HIV), hepatitis], inhibitors of DNA polymerase [Herpes Simplex Virus (HSV), human cytomegalovirus (HCMV)], protease inhibitors [hepatitis C virus (HCV), HIV], blocking of ion channel activity (influenza), and neuraminidase inhibitors (influenza, hepatitis).^17^ However, these antiviral drugs are mostly prophylactic in nature and have moderate to severe side effects. The development of antiviral drug resistance due to the high mutation rates of viruses is another major limitation. Importantly, the idea of a “universal vaccine” is of vital importance, and, therefore, new vaccine strategies are being investigated to provide a broad-spectrum of immunity against pathogens. In the last several years, biomaterials technologies together with delivery systems targeting immune cells have emerged as innovative approaches in vaccine formulations to enhance vaccine efficacy for achieving the desired immune responses.

## III. BIOMATERIALS AND NANOTECHNOLGY

Combining advancement in nanotechnology, biotechnology, and immunology, biomaterial-based vaccine formulations provide multifaceted benefits toward the development of new generation vaccines [Fig. 1(a)]. Biomaterials have distinctive physico-chemical properties of size, shape, and surface characteristics, which altogether greatly impact the efficient way of loading cargo of interest. In addition, biomaterials protect cargo from enzymatic degradation, improve stability, specifically deliver the immunogen to antigen-presenting cells (APCs), and elicit sustained release. More importantly, biomaterials enable codelivery of antigen and immune-stimulatory agents, which represent a powerful vaccination approach in the activation of immune responses.^18,19^ By far, several biomaterials have been designed to improve adjuvants and tested for infectious disease vaccination, including polymeric and inorganic particles, liposomes, virus-like particles (VLPs), and scaffolds or injectable hydrogels. Mechanisms for biomaterial-based NPs to exert their antiviral activities against infectious diseases have been discussed in many reviews.^20–22^ Organic/polymeric biomaterials have characteristic properties of biocompatibility, biodegradability, and nontoxicity, whereas inorganic biomaterials usually exhibit novel properties such as smaller particle size, high stability, tunable compositions, and high surface area and volume. In Sec. III A–E, different classes of biomaterials for vaccine trafficking, APC uptake, and successive immune response to prevent infectious diseases are discussed.

### A. Polymeric nanoparticles as adjuvants

A polymer is composed of a large molecule built from small monomeric subunits. Polymeric NPs are formulated using natural or synthetic polymers and have been investigated for vaccine formulations because of their biocompatibility, optimizable sizes and surface charge properties, and simplicity of manufacturing.^21^ Among several synthetic polymers studied for vaccine delivery, NPs prepared using poly(lactic-co-glycolic acid) (PLGA), a polymer used in the Food and Drug Administration (FDA)-approved medical devices, is a promising delivery system for antigenic proteins/peptides and adjuvants. PLGA NPs can encapsulate different biologicals, control their release kinetics, and slowly degrade over four weeks, which are critical considerations in the design of mucosal vaccines. PLGA particles have been used in several research studies for encapsulating antigens of several diseases such as hepatitis B, tuberculosis, chlamydia, malaria, leishmaniasis, toxoplasma, and allergy antigens.^22–25^ PLGA NPs help in the internalization and processing of antigens by APCs and induce a higher and sustained antibody response [Figs. 1(b)–1(d)]. Also, PLGA NPs co-encapsulating protein or peptide antigens along with rapamycin antigens were effective in eliciting a durable and antigen-specific immunological tolerance.^26^

Aside from synthetic polymers, natural biopolymer-based NPs were also found pertinent in the role of adjuvants. In recent years, plant-derived polysaccharide inulin has been formulated into delta inulin particles, known as Advax^TM^ in its adjuvant formulations. Advax^TM^ has been shown to enhance vaccine efficacy against seasonal and pandemic viral infections such as hepatitis B, influenza, pulmonary anthrax, Flaviviruses, West Nile Virus (WNV), Murray Valley encephalitis virus (MVEV), SARS, and HIV.^27^ Several chitosan-based polymeric NPs also exist for nasal vaccination. For example, crosslinked chitosan NPs have been developed to deliver recombinant hepatitis B surface antigen (rHBsAg) in a sustained and active manner against the hepatitis B virus (HBV) infections.^28^ These NPs were intramuscularly injected in mice, and the concentration of anti-HBsAg IgG produced was ninefold higher for the NPs than the conventional alum-adsorbed vaccine. In another study, a chitosan-based nanosystem was used to efficiently encapsulate fusion (F) protein gene plasmid DNA of a Newcastle Disease Virus strain with a molecular adjuvant by polyelectrolyte complexation. Nasal administration of the chitosan DNA NPs as a vaccine was highly effective in eliciting the immune response with improved lymphocyte maturation in chickens.^29^ Another biopolymer, poly-γ-glutamic acid-based NPs mixed with influenza virus hemagglutinin (HA) vaccine, was also tested in mice via intranasal administration, and sufficient cell-mediated immune responses were induced with the production of neutralizing antibody titers against influenza virus infection.^30^

### B. Liposomes/polymerosomes as adjuvants

Liposomes are spherical-shaped vesicles prepared from biodegradable phospholipids. Upon hydration, the phospholipids, either natural or synthetic, self-assemble around an aqueous core and form a lipid bilayer. Liposomes can encapsulate both hydrophobic and hydrophilic molecules, such as entrapment of water-soluble antigenic proteins, peptides, and nucleic acids within the aqueous core of liposomes, whereas adsorption or chemical binding of lipophilic antigens or adjuvants into the lipid bilayer.^31^ In recent years, liposome formulations have been used as therapeutic vaccines against infectious diseases and are approved clinically.^32–34^ For example, Epaxal^®^ virosomes containing antigen for hepatitis A viral strain with lipid components of dioleoylphosphatidylethanolamine (DOPE) and 1,2-dioleoyl-sn-glycero-3-phosphocholine (DOPC) have demonstrated successful immunogenicity against hepatitis A virus (HAV) infection. Another vaccine, Inflexal™ V virosomes, containing hemagglutinin (HA) of influenza A and B virus strains within structural components of lecithin and cephalin, showed statistically significant immunogenicity against influenza than other subunit vaccines. Additionally, a new class of liposomal drug carrier systems, interbilayer-crosslinked multilamellar vesicles (ICMVs), has shown stronger humoral and cellular immunity in preclinical models of malaria^23^ [Figs. 1(e)–1(f)], hepatitis C,^35^ and Ebola.^36^

### C. Virus-like particles as adjuvants

Spherical-shaped VLPs are self-assembled viral capsid proteins in the size range of 20–200 nm in diameter. These particles are produced from proteins obtained by cloning and expression of the gene of interest in culture using host systems such as bacteria, yeast, insects, plants, and mammals. VLPs are noninfectious, carry no genetic materials, mimic viral structure, and have very similar antigenic epitopes analogous to native viruses that facilitate uptake by APCs and subsequent activation.^37,38^ VLP-based vaccines are also validated with the clinical success of Engerix for HBV and Cervavix for human papillomavirus (HPV) (both from GlaxoSmithKline) and Recombivax HB for HBV and Gardasil for HPV (both from Merck and Co., Inc.).^39^

### D. Inorganic nanoparticles as adjuvants

Several synthetic inorganic NPs have been successfully formulated as vaccine delivery systems because of their appropriate physiochemical properties, such as ease of synthesis, tunable particle sizes and shapes, stability, high porosity, high surface-to-volume ratio, and favor the conjugation of biomolecules. Gold (Au), silver (Ag), silica (SiO_2_), carbon NPs, carbon nanotubes (CNTs), ferric oxide (Fe_2_O_3_), and calcium phosphate (CaP) are well-known adjuvants *per se*, which can be easily fabricated into variable shapes and sizes to induce a stronger cellular and humoral immune response against viral infections.

AuNPs are one of the most extensively used inorganic particles in designing vaccine delivery systems.^40^ AuNPs synthesized with different shapes (such as nanospheres and nanorods) have a high affinity for thiol groups, which allow easy coupling with thiol-modified proteins, peptides, and oligonucleotides. AuNPs functionalized with biomolecules are readily internalized by APCs and induce the desired immune response. AuNP-based vaccines by conjugating with adjuvants and respective antigens have been developed for influenza, foot and mouth disease (FMD), cancer, malaria, and HIV.^41–45^

Mesoporous silica NPs (MSNPs) are another highly promising material for developing vaccine delivery systems because of their mesoporous structure, favorable chemical properties, thermal stability, and biocompatibility. Unlike solid silica and other NPs, MSNs have a tunable pore size (2–50 nm), larger pore volume, high surface area, ordered structure, and high mechanical strength, which are highly suitable for encapsulation of vaccine antigens. MSNPs in the size range of 50–200 nm have been demonstrated as antigen carriers and adjuvants for the effective delivery of antigens to APCs.^46–48^ For example, as vaccine carriers, hollow mesoporous silica nanoparticles (HMSNPs) loaded with porcine circovirus type 2 (PCV2-ORF2) proteins elicit specific antibodies and cell-mediated immune responses with no cytotoxic effects in mice.^49^ Also, amino‐functionalized Mobil Composition of Matter No. 41 (MCM-41) as a carrier for antigenic ovalbumin (OVA) protein was investigated for immunization in mice.^50^

In recent years, carbon-based nanomaterials such as carbon nanotubes and graphene have gained attention in the field of nanomedicine as well as in immunotherapy. Specifically, CNTs are promising because they are intrinsically non-immunogenic, are less toxic, can be encapsulated with multiple antigens, are rapidly taken up by APCs, and boost the efficacy of antigens.^51–53^ CNTs conjugated with antigen peptide from the foot-and-mouth disease virus (FMDV) were injected into mice that resulted in an immunogenic response by eliciting virus-neutralizing antibodies.^54^ Graphene oxide (GO) and graphene quantum dots (GQDs) have also been investigated as HIV inhibitors^55,56^ and carbon nanodots for Herpes Simplex Virus (HSV) inhibitors.^57^

Several other inorganic NP-based vaccine formulations have also been studied for treatment and immunization against infectious diseases. Recently, calcium phosphate NPs (CaPs) have been used as biodegradable antigenic carriers. In a novel immunization approach, biodegradable CaP NPs have been used to deliver TLR9 ligand (CpG) in combination with an antigen from the influenza A virus (hemagglutinin). In mice, uptake of these functionalized CaPs (unmethylated cytosine–guanine dinucleotide) by dendritic cells (DCs) was highly efficient and induced a potent immune response against influenza virus mediated by T cells.^58^ Advances have also been made in iron oxide NP (IONP)-based vaccine adjuvants. To increase the vaccine potency, IONPs coated with mannose and HBsAg have been used to target receptors on DCs that showed a positive effect with higher immune responses in mice.^59^

### E. Biomaterial scaffolds for vaccine delivery

Following recent novel approaches in biomaterial-based immunotherapy, scaffolds made of synthetic and natural materials also have great potential in modulating immune responses against infectious diseases. Recent findings and emerging scaffold-based strategies for cancer immunotherapy, chronic infections, autoimmune disorders, and systems for immune microenvironments have been extensively discussed in the cited reviews.^60–62^ Importantly, scaffolds and hydrogel-based scaffolds can easily encapsulate antigens and immunomodulators and mimic the immunogenicity of natural infection environments by recruiting and activating APCs. In an approach to reduce the repetitive vaccine injections, the hepatitis B vaccine (Engerix-B16) was encapsulated into a polyethylene glycol PEG-based hydrogel depot. The hydrogel was designed to be stimulus responsive to the clinically approved novobiocin. Subcutaneous implantation of this hydrogel in mice and the release of vaccine by the oral administration of novobiocin demonstrated successful immunization in mice with significantly higher anti-HBs titers as compared to control mice without novobiocin.^63^ In another study, for prolonged active immunomodulation against influenza A, a novel injectable pathogen‐mimicking hydrogel (iPMH) has been proposed for improving both the cellular and humoral immune responses. Injectable iPMH hydrogels were prepared by combining *in situ* gel‐forming collagens with pathogen‐mimicking adjuvants, which are complexes of poly(γ‐glutamic acid) with abundant carboxylate groups and dispersion helper function, hydrophobic immunostimulatory 3‐O‐desacyl‐4′‐monophosphoryl lipid A (MPLA) molecules, and amphiphilic protein antigens [ovalbumin (OVA) and viral antigens]. When mice were immunized with iPMH(OVA), it induced high levels of antigen‐specific IgG titers and IFN‐γ‐producing cells, exhibiting complete protective immunity against lethal challenge of the infectious 2009 H1N1 and the highly pathogenic 2006 H5N1 (Fig. 2).^64^ More recently, polymer-nanoparticle (PNP)-based injectable and self-healing hydrogels have been introduced as effective delivery platforms for the sustained release of subunit vaccines.^65^ In this study, aqueous solutions of hydroxypropylmethylcellulose derivatives (HPMC−C12) were mixed with polymeric NPs of poly(ethylene glycol)-b-poly(lactic acid) (PEG−PLA) to form self-assembled PNP hydrogel, which was later encapsulated with a model vaccine containing OVA and Poly(I:C) and administered in mice for an *in vivo* study. This vaccine loaded PNP hydrogel platform demonstrated that the sustained release of antigens raises the extent and timescale of the responses of germinal centers (GCs) in the lymph nodes that ultimately enhances the potency of humoral immune response.^65^ Therefore, these hydrogel-based scaffolds present new opportunities for the development of injectable scaffolds for local immunization and vaccine delivery. However, preclinical testing in animals and humans needs to be conducted in the future.

### F. Challenges and perspectives for biomaterial-based adjuvants

Although nanoparticles are excellent supports for loading and simultaneous co-delivering different antigens and proteins to synergistically elicit an enhanced immune response, several challenges remain unaddressed. These bottlenecks include the control of physical and chemical properties (core chemistry, size, shape, and surface properties), scale-up and reproducibility of synthesis, controlled interactions with biological systems, and minimizing undesired side-effects on cells, tissues, and organs. Systematic research on immunotherapy with detailed studies on suppression, activation, and stimulation of immune responses in relation to NP properties will help in the successful clinical translation of particle-based-vaccines against deadly diseases. Most of the studies also lack *in vivo* experiments, which is the primary step toward human clinical trials. Therefore, we need to develop a deeper understanding of the impact of NPs on human health before large-scale production and their application in immunization.

As mentioned above, hydrogel-based vaccines and immunotherapies have also shown enormous promise in improving public health; however, most of these reported attempts have data only on *in vitro* cell culture models and *in vivo* animal models,^61,62^ and therefore, these vaccines need further validation for human use. Recently, platforms that better recapitulate human physiology *in vitro* than conventional culture are being engineered as alternatives to animal models. Toward this end, tissue engineering techniques and 3D printing in combination with microfluidics could be used to construct the organs and immune tissues, such as thymus, lung, bone marrow, spleen, lymph nodes, and their intracellular compartments, as well as infection models to study human immune function and diseases.^62,66,67^ One of these engineered platforms, organ-on-a-chip technology, has allowed to recapitulate the life cycle of HBV in a 3D microfluidic primary human hepatocyte (PHH) culture.^68^ Different viral infection models could be engineered similarly to study the pathophysiological characteristics of human viral infections and to test drug candidates.^69^

## IV. NATURAL PRODUCTS AGAINST VIRAL INFECTIONS

Natural products are also an excellent source of biological materials for discovering novel antiviral therapeutics. In recent years, several reviews have been published on traditional medicine and the discovery of natural product-based therapeutics against many viral infectious diseases.^70–76^ Interestingly, a substantial number of research studies on medicinal plants from Indian (Ayurveda), traditional Chinese medicine (TCM), Unani, Chakma medicines, and many other systems have served as potential alternative treatments to reduce the impact or severity of diseases caused by pathogenic viruses.^77–79^ Several active phytochemicals are known to have therapeutic effects against genetically and functionally diverse viral families. These phytochemicals include the flavonoids, organosulfur compounds, polyunsaturated fatty acids, vitamins, phytoalexins, lignans, polyphenolics, quinones, lactoferrin, tannins, limonoids, sulfides, coumarins, furyl compounds, saponins, polyines, thiophenes, chlorophyllins, alkaloid proteins, and peptides and are described in several recent reviews.^70–74^ The antiviral mechanism of different natural products and formulations has been elucidated by means of their antioxidant activities, inhibiting various stages of the viral life cycle, inhibiting DNA and RNA synthesis, inhibiting the viral reproduction, etc.^71,77,80^

Large numbers of herbal medicinal products have been studied for their immunomodulatory and antimicrobial bioactivities by *in vitro* and *in vivo* bioassays.^71,74^ Out of 100 British Colombian medicinal plants that have been investigated for antiviral activity, a few of them showed antiviral activities against coronaviruses, HSV type 1, parainfluenza, respiratory syncytial virus (RSV), and rotavirus.^81^ Sulfated polysaccharide groups that were extracted from algae and cyanobacteria are reported to show activity against HIV and HSV.^82^ Also, multiple herbs and different herbal formulations have been studied against viral infections, such as measles viruses, human rotaviruses (HRVs), RSV, human rhinoviruses, the coxsackie group of viruses, neurotropic Sindbis virus (NSV), and various strains of poliovirus.^83^

Although a significant number of natural products have been reported as potential antiviral agents, additional work is still needed. Further characterization of herbal formulations, pharmacological profiling, and authentication of medicinal plants for human consumption may contribute to the eradication of complicated viral infection. More clarification and clinical data are required as most of the antiviral behavior of natural products in animal testing and their utilization as antiviral therapeutics is still in the early stage. In a novel approach for antiviral drug discovery, biomaterials in the form of nanoparticles, hydrogels, and scaffolds could be used for the targeted delivery of phytochemicals through sustained or stimulus-responsive platforms.^84–86^ Additionally, computational and artificial intelligence (AI)-based models would help in the virtual screening of phytochemicals to select potential antiviral drug candidates.^87^ Also, plenty of research has been done for the delivery of phytochemicals in cancer treatment that could be adapted for developing antiviral therapies.^88^

## V. ANTIVIRAL SURFACES

Despite the fact that viruses cannot replicate on surfaces, pathogen-contaminated surfaces (fomites) are believed to be important in the transmission of respiratory viral infections as well as the cause of disease outbreaks.^89,90^ With all decades of effort, there are still no vaccines against global pandemics; therefore, the severity of outbreaks such as the transmission of RSV via fomites needs to be prevented through hygienic practices and different control measures.^91,92^ Recently, several disinfection alternative technologies have been developed to prevent contamination and subsequent viral propagation on surfaces, such as the application of aerosolized hydrogen peroxide, UV light, high-intensity narrow-spectrum light, and cold plasma technology.^93,94^ However, these no-touch technologies as well as conventional cleaning methods using sodium hypochlorite and disinfectants containing 70%–85% ethanol still present several limitations, including laborious cleaning and time. In recent years, many conventional and advanced techniques for surface modification have been utilized for fabricating contact-active antimicrobial surfaces.^95–97^ Antiviral surfaces can be fabricated by either physical or chemical treatments, broadly categorized as surface architecture modification, polymerization, functionalization, derivatization, and encapsulation of nanomaterials. These are emerging technologies for self-disinfecting surfaces to control the spreading of infections with more products upcoming. Some recent results and evolved strategies to impart long-lasting anti-adhesive and antiviral properties to surfaces are discussed below and summarized in Table I.

**TABLE I.** Summary of different antiviral surfaces.

Antiviral materials	Surface features	Viruses	Incubation time	Result
Al 6063 alloy surfaces	Nanostructured surfaces, 23 nm ± 2 nm	Respiratory syncytial virus (RSV), rhinovirus (RV)	24 h	More efficient against RV than RSV, 3 − 4 log10 reduction observed in viable virus.^100^
Al 6063 alloy surfaces	Nanostructured surfaces, 23 nm ± 2 nm	Severe acute respiratory syndrome coronavirus 2 (SARS-CoV-2)	6 h	Antiviral activity, 5 log reduction with no recoverable viable virus.^101^
PTFE nanoparticles thermally sintered to PP microfibers	Superhydrophobic low surface energy, multilength scale roughened textiles	Adenovirus type 4 and 7a	–	Reduced the attachment of HAdv4 and HAdv7a virions by 99.2 ± 0.2% and 97.6 ± 0.1%, respectively.^102^
Polyethylenimines (PEIs) derivatives	Hydrophobic polycationic coatings	Influenza (H1N1) virus	30 min	Killed influenza virus with 100% efficiency, at least a 4-log reduction in the viral titer.^13^
PEIs	Hydrophobic polycationic coatings	Poliovirus, rotavirus	30 min	Disinfected aqueous solutions of enveloped and non-enveloped viruses.^105^
QACs	Hyperbranched polymers with quaternary amines	Poliovirus Sabin1, influenza A (H1N1) virus	1 h	Virucidal activity only against enveloped influenza A (H1N1) virus.^106^
QBEst, QBAm	Cationic polymeric coatings, zwitterionic structure	Influenza (H1N1) virus	30 min	QBEst and QBAm coated surfaces reduced the viral burden by >1000- and >10 000-fold.^107^
Copper	CuO NPs impregnated into N95 masks	Human influenza A virus (H1N1), avian influenza virus	30 min	Potent anti-influenza biocidal properties.^114^
Silver	PHBV films coated with AgNP fibers	FCV, MNV	150 days	No infectious FCV, MNV titers decreased by 0.86 log.^116^

### A. Anti-adhesive surfaces

Over the past few years, physical and chemical modification strategies have been advanced to achieve surfaces having anti-adhesive properties against microorganisms. Fabrication of these surfaces has been inspired by the excellent anti-adhesive structures found on natural surfaces of insect wings, marine organisms, gecko foot, and lotus leaf.^98,99^ The presence of micro- and nanoscale architectures on the insect wings allows antimicrobial properties. Similarly, biomaterial surfaces with suitable topographical features for controlling cell adhesion can be fabricated using physical modification techniques such as photolithography, demixing, dewetting, physical vapor deposition, laser surface modification, sand blasting, and ion beam assisted deposition. Recently, the antiviral activity of aluminum (Al) 6063 alloy surfaces with randomly aligned ridges was investigated against common respiratory viruses (RSVs), rhinovirus (RV), and SARS-CoV-2.^100,101^ These nanostructured surfaces with excellent nanomechanical properties were effective in reducing the surface contact transmission of both enveloped and non-enveloped viruses and, importantly, SARS-CoV-2 **[** Figs. 3(a)–3(c)].

Lotus leaf has self-cleaning nature due to its superhydrophobic surface. The hierarchical topography with a waxy cuticle keeps lotus leaf water repellent as well as inhibits the settlement of microorganisms or any contaminants.^99^ Therefore, reducing the adhesion of virions via chemically modified surfaces with self-cleaning superhydrophobic properties is also an interesting approach, which needs to be studied thoroughly and systematically. Recently, nonwoven polypropylene textiles treated with polytetrafluoroethylene (PTFE) NPs followed by thermally sintered to superhydrophobic polypropylene (PP) microfibers have been shown to reduce the attachment of non-enveloped viruses, adenovirus types 4 and 7a by 99.2 ± 0.2% and 97.6 ± 0.1% (2.10 and 1.62 log), respectively, compared to non-coated controls [Figs. 3(d)–3(f)].^102^

### B. Surface functionalization

Functionalization of surfaces is the most common approach to obtain antiviral properties on different materials. The surface functionalization method involves the inclusion of antiviral entities or chemical compounds on various material surfaces to reduce the viability of viruses. This includes the incorporation of long-chain, hydrophobic, positively charged coatings such as alkylated PEIs or functional groups such as quaternary ammonium and phosphonium cations, which impart antiviral properties to the surface^13^ [Figs. 4(a)–4(c)]. For example, specifically designed hydrophobic polycations of PEI derivatives, either attached covalently or physically deposited onto glass surfaces, have been shown to rapidly kill the influenza virus within minutes and efficiently inactivated several other viruses such as the drug-resistant strains of influenza virus, rotavirus, and poliovirus.^13,103–105^ Here, the virucidal action appears to be similar to that of porcupine needles made up of rigid and erect hydrophobic polycationic chains that rupture the viral lipid envelopes on contact.^103^ Likewise, plastic and glass surfaces coated with quaternary ammonium compounds (QACs) were virucidal against the enveloped influenza A (H1N1) virus by disrupting the viral envelope within 2 min.^106^ Recently, polyurethane sheets coated with the quaternary benzophenone-based ester (QBEst) and quaternary benzophenone-based amide (QBAm) exhibited excellent activity against the influenza virus with 100% killing on 30 min of treatment.^107^

### C. Metal and nanomaterials for antiviral activity

Materials with intrinsic antimicrobial properties are an innovation in designing antimicrobial surfaces. These materials, such as silver, copper, and zinc, and polymers such as chitosan have a natural ability to kill or eliminate pathogenic microorganisms [Figs. 4(d)–4(f)]. Recent effort and research in nanotechnology have demonstrated the potential of metal ions, metal compounds, and metal NPs in controlling various pathogenic microorganisms. Silver NPs have been studied for their effective antiviral activity against HIV-1, RSV, hepatitis B virus, HSV type 1, influenza virus, monkeypox virus, and Tacaribe virus and are thoroughly reviewed in the cited reference.^108^ Copper also has the potential to destroy viruses, such as influenza viruses, murine norovirus (MNV‐1), human immunodeficiency virus (HIV), and coronaviruses.^109^ Antiviral efficacy of zinc has also been studied against measles virus, influenza, HIV, HSV, RV, HCV, coxsackie virus, and many other viruses.^110–112^ Compounds of zinc, cadmium, and mercury compounds have shown anti-HIV-1 activities.^113^ However, most of these studies have been dedicated to the treatment, and further research needs to be focused on limiting the spread of the virus during outbreaks, such as the application of NPs and metal salts in the development of safe and more effective personal protective equipment (PPE).

Over recent years, some publications have been reported on well-known antimicrobial formulations and nanostructures containing copper, silver, and zinc species to prevent and limit both contamination and contagion. For example, in the preparation of antiviral respiratory protective face masks, CuO NPs impregnated into disposable N95 respirator masks were reported to exhibit high anti-influenza biocidal properties without changing their physical barrier properties.^114^ The antiviral activity of nanosized CuI particles against pandemic (H1N1) 2009 influenza virus were also investigated and proposed for their application in antiviral filters, face masks, protective clothing, and kitchen cloths.^115^ Silver nitrate and silver NPs were evaluated for inactivating norovirus surrogates, i.e., feline calicivirus (FCV) and MNV. In addition, poly (3-hydroxybutyrate-co-3-hydroxyvalerate) (PHBV) films were coated with PHBV18/AgNP fiber mats to develop virucidal biopolymers/coatings that may be suitable as an active material, particularly in contact surfaces in food and medical industries^116^ [Figs. 4(d)–4(f)].

## VI. CHALLENGES AND PERSPECTIVE FOR BIOMATERIAL-BASED ANTIVIRAL SURFACES

Although different approaches and techniques have been used in designing antiviral surfaces as well as their promising antiviral results were demonstrated, these strategies have certain drawbacks. Importantly, more studies and a systematic approach in testing the cost-effectiveness, durability, and stability of such materials and coatings are required. Although recent studies show that engineering nanoscale topography on metallic surfaces minimizes the attachment of virions, extensive scientific research studies related to topography parameters at the micro-/nano- level need to be done for the universal design of an antiviral model. The adhesion force measurement, Engineered Roughness Index (ERI), size, the spacing of contours, and dimensions of topographic features are some of the important parameters, which may help in establishing a universal model for surface topography to inhibit the attachment and growth of pathogenic viruses. These antiviral studies have mostly been limited to short-term *in vitro* environments, whereas, during clinical use to minimize nosocomial infections, the microbicidal action may drastically diminish with the deposition of cell debris, dirt, and grease. Thus, economical and technically feasible solutions to recover the antimicrobial properties such as cationic detergents or cationic spraying solutions need to be developed. Also, the inclusion of polymers with anionic and cationic functional groups contributes to broad-spectrum antipathogenic activity. For example, anionic carbosilane dendrimers bearing carboxylate, naphthylsulfonate, and sulfate terminal groups have shown powerful antiviral activity against HIV infection^117^ while pyridinium-type polycations against the influenza virus.^118^ Furthermore, much additional work is required to confirm the stability and human safety of antimicrobial solutions, polymers, and others before widespread adoption in hospital settings and others.

## VII. APPLICATION OF BIOMATERIALS AGAINST RECENT OUTBREAKS AND PERSPECTIVES

The ongoing coronavirus disease (COVID-19) pandemic that started in December 2019 with a few patients of Wuhan Province, China, has now spread worldwide in 2020 and resulted in millions of deaths around the world. COVID-19 caused by the zoonotic transmission of the virus SARS-CoV-2 is closely related to previous epidemics of SARS coronavirus (SARS-CoV) and Middle East respiratory syndrome coronavirus (MERS-CoV) in 2002 and 2012, respectively. In view of the current pandemic and possible future outbreaks, biomaterial technologies need to be further explored to control and manage infectious diseases. These approaches include biomaterial-based vaccine delivery systems, disinfectants, antiviral surface coatings, antiviral face masks and accessories, and so on. Below, we have discussed some selected biomaterial-based technologies that have been developed in response to COVID-19 pandemic.

### A. Vaccine delivery systems

An effective vaccine is urgently required to halt Covid-19 pandemic. Therefore, tremendous effort has been put into ongoing research to develop effective and safe vaccines against SARS-CoV-2.^119,120^ As per draft landscape of COVID-19 candidate vaccine, prepared by the World Health Organization (WHO) on 12 November 2020, and available at https://www.who.int/publications/m/item/draft-landscape-of-covid-19-candidate-vaccines, 48 candidate vaccines are in clinical evaluation and 164 candidate vaccines are in preclinical evaluation. These vaccines are based on inactivated vaccines, recombinant protein vaccines, vectored vaccines, and RNA- and DNA-based vaccines.

Several biomaterial-based solutions such as nanoparticles, virus-like particles, scaffolds, and microneedles can be put forward against SARS-CoV-2. Specifically designed biomimetic materials can be used to protect multiple antigen cargos, and further surface antibody conjugation allows specific immune cell or tissue targeting.^121^ Microneedle‐based polio, influenza, and hepatitis B vaccines have been developed earlier for pain‐free antigen delivery into the skin, which is a highly immune‐reactive organ [Fig. 5(a)]. Utilizing a similar approach, a polymeric microneedle array‐based MERS vaccine using a MERS‐CoV S protein subunit trimer as an antigen has been developed, and the results confirm a sustained release of antigen‐specific antibody responses in mice. Furthermore, the MERS vaccine has been modified and designed to produce a similar microneedle vaccine based on SARS‐CoV‐2 S protein subunit trimers, which shows similar immune responses in mice.^122^ In another approach, the ability of a biomaterial-based vaccine using mesoporous silica rods (MSRs) has been demonstrated. Upon subcutaneous injection, these rods spontaneously assembled into a 3D macroporous structure and were able to generate humoral responses against SARS-CoV-2 relevant antigens.^123^

### B. Lung models

High mortality rates associated with Covid-19 pandemic are ascribed to the diffuse alveolar damage (DAD) that eventually cause acute respiratory distress syndrome (ARDS) in COVID-19 patients. To further understand the coronavirus mechanism and screening of drugs and vaccines against infection, 3D human tissue-like models are acutely needed. Recent developments in stem cell-derived 3D cell cultures, widely referred to as organoids, have enabled the generation of physiologically relevant human tissue/organ models.^124^ For example, alveolosphere cultures of primary human alveolar epithelial type-2 cells (AT2s) have been developed into lung organoids or mini lungs that mimics human lung tissues for *ex vivo* studies [Fig. 5(b)].^125^ These mini lungs when infected with SARS-CoV-2 demonstrated virus replication, inflammatory responses of IFN, surfactant dysfunction, and cell death. In the other experiment, the prophylactic effectiveness of IFNs against SARS-CoV-2 was demonstrated by pre-infection treatment with low doses of interferons that slow down the viral replication. The development of such mini lungs provides a research model to examine lung responses during infection as well as pathological changes and preclinical testing of drug toxicity and reactions. However, these mini lungs have limitations, and further studies related to age differences of donors, tissue composition, and healthy donor vs immunocompromised donor need to be investigated.

### C. Antiviral face masks and accessories

To reduce the nosocomial transmission of coronavirus, various protecting kits have been prepared using thermoplastic polymers because of their resistance properties of heat, wear, and chemical. Sheets of polycarbonate (PC) and poly(ethylene terephthalate) (PET) are used to prepare surgical face shields, whereas polypropylene is used for certified masks such as N95 Filtering Facepiece against Particles FFP2 and FFP3.^126–128^ However, as the COVID-19 pandemic is prolonged and continues to spread unabated, the demand for personal protective equipment (PPE) is high to protect the frontline healthcare workers and has led to a worldwide shortage of face masks and raw materials. Also, PPE in landfills has raised environmental concerns due to improper disposal of PPE.^129^ Therefore, further research needs to focus on the development of PPE using biodegradable polymers, bioplastic, and natural biopolymers after increasing their thermal stability. Recently, a fully compostable and biodegradable medical N95 mask, named Can-Mask, has been made using wood fibers of pine, spruce, cedar, and other softwoods.^130^ Also, performance of PPE can be improved with incorporation of antimicrobial metal nanoparticles that will allow self-sterilization and can be re-used. For example, facile FFP3 mask sputter coated with silver nanocluster silica composite showed a virucidal effect toward SARS-CoV-2 [Fig. 5(c)].^131^

### D. Antiviral coatings

COVID-19 is highly contagious and easily spreads through aerosolized droplets of SARS-CoV-2 deposited on surfaces, and the virus can remain active for several days.^132^ Frequent cleaning of contaminated surfaces using disinfectant in all public spaces, hospitals, and private places is a major challenge. Biomaterial-based antiviral coatings made from metal NPs, cationic surfactants, and natural products are a promising technique to limit both contagion and contamination of COVID-19 [Fig. 5(d)]. Nanoparticles such as Ag, Au, zinc oxide (ZnO), titanium dioxide (TiO_2_), and copper oxide (Cu_2_O) are commonly used antiviral agents to inactivate the viruses. For example, during the COVID-19 pandemic, to self-sterilize the buildings in Milan, a disinfectant formulation based on titanium dioxide and silver nanoparticles was used for cleaning.^133^ Recently, Cu_2_O particles bound with polyurethane (PU) coatings were applied on different surfaces that showed 99.9% reduction of SARS-CoV-2 titer within 1 h.^134^ Furthermore, UV radiation and heating approaches are effective at killing SARS-CoV-2; so, the incorporation of coating materials with thermo-optical properties could also be of great importance for instant killing of viruses on the surfaces.

## VIII. CONCLUSION

The catastrophic spike in viral infections and high mortality rates motivated the need for new antiviral therapeutics and preventive strategies to combat viral infections. We have herein reviewed major progress in biomaterial approaches in antiviral therapeutics and anti-infective surfaces to combat different viral infections. In recent years, biomaterials in the forms of NPs have been described as excellent candidates in the development of antiviral vaccines and applied to design new vaccine carriers to target specific viruses and infection pathways. A myriad of antiviral formulations prepared with polymeric NPs, liposomes, VLPs, and inorganic NPs have shown significantly higher activity than that of free antigens, proteins, or peptides. Overall, these biomaterial-based vaccine delivery systems have several merits, such as biocompatibility, high antigen loading, and stabilization, protect premature degradation and pathogen characteristics, promote uptake by APCs and controlled release of antigens, and are suitable for intranasal administration.

Recent advancements and experimental scaffold-based vaccines in clinical trials have shown potential benefits in prophylactic and therapeutic vaccination. Hydrogel-based vaccine depots enable the codelivery of vaccine components locally to initiate the vaccine response through recruitment of APCs, while providing sustained release of the vaccine cargo. This strategy has been primarily applied for cancer immunotherapy by codelivering patient-derived tumor lysates and immunomodulatory agents for innate immune activation, and further research needs to be focused on controlled release vaccine delivery systems that could also enhance humoral immune responses.

The currently available antiviral drugs/vaccines, though effective, are beyond the reach of common people in developing countries. As life-threatening incurable virus species are continuously increasing with high mortality rates, an alternative affordable antiviral approach of natural compounds and products to inhibit the different pathways involved in the virus replication cycle will help in slowing down the viral infection in humans.

Fomites remain an important mechanism of transmission of many microbes. We also reviewed recent research on physical and chemical functionalization strategies for designing surfaces that reduce the adhesion of virions. Major advances include modifying the topographical structure of the surfaces and functionalization of surfaces with polycations and surfaces with inorganic dopants.

In the coming years, certainly, we will see more epidemics/pandemics. We believe that new strategies and biomaterials science will help us in intensifying our abilities to control future viral outbreaks and research in this field must be vigorously pursued.

## Data Availability

Data sharing is not applicable to this article as no new data were created or analyzed in this study.
